# Early neuropathological and neurobehavioral consequences of preterm birth in a rabbit model

**DOI:** 10.1038/s41598-019-39922-8

**Published:** 2019-03-05

**Authors:** Johannes van der Merwe, Lennart van der Veeken, Sebastiano Ferraris, Willy Gsell, Uwe Himmelreich, Jaan Toelen, Sebastien Ourselin, Andrew Melbourne, Tom Vercauteren, Jan Deprest

**Affiliations:** 1 Department of Development and Regeneration, Cluster Woman and Child, Group Biomedical Sciences, KU Leuven University of Leuven, Leuven, Belgium; 2 Department of Obstetrics and Gynaecology, Division Woman and Child, University Hospitals Leuven, Leuven, Belgium; 3 Translational Imaging Group, Centre for Medical Image Computing (CMIC), Department of Medical Physics and Biomedical Engineering, University College London, London, UK; 4 moSAIC facility, Biomedical MRI, Department of Imaging and Pathology, KU Leuven, Leuven, Belgium; 5 Department of Pediatrics, Division Woman and Child, University Hospitals Leuven, Leuven, Belgium; 6 School of Biomedical Engineering and Imaging Sciences, King’s College London, London, UK; 7 Institute for Women’s Health, University College London, London, UK

**Keywords:** Encephalopathy, Neonatal brain damage

## Abstract

Preterm birth is the most significant problem in contemporary obstetrics accounting for 5–18% of worldwide deliveries. Encephalopathy of prematurity encompasses the multifaceted diffuse brain injury resulting from preterm birth. Current animal models exploring the underlying pathophysiology of encephalopathy of prematurity employ significant insults to generate gross central nervous system abnormalities. To date the exclusive effect of prematurity was only studied in a non-human primate model. Therefore, we aimed to develop a representative encephalopathy of prematurity small animal model only dependent on preterm birth. Time mated New-Zealand white rabbit does were either delivered on 28 (pre-term) or 31 (term) postconceptional days by caesarean section. Neonatal rabbits underwent neurobehavioral evaluation on 32 days post conception and then were transcardially perfuse fixed. Neuropathological assessments for neuron and oligodendrocyte quantification, astrogliosis, apoptosis and cellular proliferation were performed. Lastly, *ex-vivo* high-resolution Magnetic Resonance Imaging was used to calculate T1 volumetric and Diffusion Tensor Imaging derived fractional anisotropy and mean diffusivity. Preterm birth was associated with a motoric (posture instability, abnormal gait and decreased locomotion) and partial sensory (less pain responsiveness and failing righting reflex) deficits that coincided with global lower neuron densities, less oligodendrocyte precursors, increased apoptosis and less proliferation. These region-specific histological changes corresponded with Magnetic Resonance Diffusion Tensor Imaging differences. The most significant differences were seen in the hippocampus, caudate nucleus and thalamus of the preterm rabbits. In conclusion this model of preterm birth, in the absence of any other contributory events, resulted in measurable neurobehavioral deficits with associated brain structural and Magnetic Resonance Diffusion Tensor Imaging findings.

## Introduction

Preterm birth (PTB), <37 completed weeks of gestation, is the most significant clinical problem in contemporary obstetrics accounting for 5–18% of worldwide deliveries^1^. Advances in perinatal medicine have resulted in earlier prediction and more timely interventions leading to increased neonatal survival rates, yet the associated morbidity in survivors remains significant. Particularly neurocognitive sequelae are common after both early, at <34 weeks’ gestation, and late PTB, at 34–36 weeks’ gestation^2,3^. The term ‘encephalopathy of prematurity’ (EoP) has been proposed to define the typical brain injury and resulting neurological sequelae of PTB^4^. EoP incorporates diffuse white matter injuries (WMIs) with specific lesions to the thalamus, basal ganglia, cerebral cortices, brainstem and cerebellum^5^.

Animal models are crucial for the appraisal of neurodevelopmental processes and several species have been used to clarify the underlying pathophysiology of EoP. No ideal model currently exists as brain development across species is not equal at birth^6^. Furthermore, most models employ one out of a range of significant insults (mainly mediated either by hypoxic-ischemic or infective/inflammatory insults) to generate gross abnormalities^7^. These insults mimic different etiologies of PTB which may be involved, either alone or in combination with prematurity. The exclusive effect of prematurity was firstly described in context of general health and activity outcomes^8–11^. While neurodevelopmental specific outcomes were reported in a non-human primate model^12–14^ and in one guinea pig model^15^ that primarily reported on neuropathological outcomes.

Lately, the rabbit has been widely employed for modeling brain damage after perinatal injury in models of cerebral palsy^16,17^, hypoxia-ischemia^18^, intraventricular hemorrhage^19,20^ and intrauterine infection^21–23^. The rabbit seems best suited to bridge the gap between small and large animals being widely available, having low housing needs, a short reproductive cycle, timed gestation with large litters and placental development close to human^24,25^. In translational neuroscience rabbits have the advantage of a more complex brain structure with greater white matter proportion than rodents and the timing of perinatal brain white matter maturation is comparable to the human^17,26^. Our group has recently published a MR multi-atlas for the neonatal rabbit brain with an automatic segmentation propagation and label fusion algorithm^27^ facilitating comprehensive MRI image analysis.

Herein we aimed to define the early neurodevelopmental consequence of iatrogenic prematurity in a rabbit model, uncomplicated by perinatal infection, hypoxia-ischemia or any other insult. Prematurity resulted in distinctive neurohistological, DTI and early neurobehavioral changes at a term corrected equivalent age.

## Results

On postconceptional age (PCA) 32 days, 36 PCA 28 day and 84 PCA 31 day rabbits were evaluated. Born from 14 and 12 does respectively, with comparable litter sizes (10 ± 2 vs. 10 ± 3), thus being four (PCA 28d) and one (PCA 31d) day after birth. Further delivery details of the pregnant does are provided in Supplementary Fig. 4.

### PTB is associated with a motoric and partial sensory deficit

**Figure 1:**
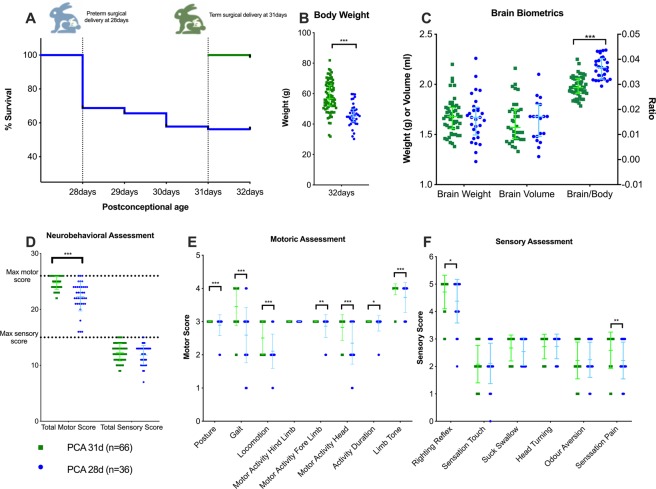
Rabbits were delivered at either postconceptional age (PCA) 28 or 31 days. New-born rabbit survival (**A**), body weight (**B**) and brain biometrics (**C**) on PCA 32 days. Neurobehavioral total sores (**D**) with individual motor (**E**) and scenery (**F**) assessments illustrated. PCA 31d rabbits = 66; PCA 28d rabbits = 36. Data displayed as median and IQR with significance as *0.05 ≥ p > 0.01; **0.01 ≥ p > 0.001; ***p < 0.001.

PTB was associated with a significant mortality of 44% (28/64) vs. 0/84 in the PCA 31d group. By 32 postconceptional days, the PCA 28d rabbits weighed less [45.3 g (41.5–49.6) vs. 56.9 g (51.9–65.3); p < 0.001] but had similar brain weights [1.67 g (1.52–1.83) vs. 1.68 g (1.56–1.77); p = 0.58] and brain volumes [1.68 mL (1.48–1.80) vs. 1.57 mL (1.46–1.75); p = 0.91], resulting in a significant higher brain/body ratio [0.0370 (0.0335–0.0409) vs. 0.0287 (0.0264–0.0324); p < 0.001]. (Fig. 1). Supplementary Fig. 4 illustrates weight gain during the postnatal period.

Preterm rabbits had a significant motoric deficit [motor score 23 (21–24) vs. 25 (24–26); p < 0.001]. The motor deficit was mainly due to posture instability (p = 0.003), abnormal gait (p < 0.001) and a decrease in total locomotion (p < 0.001). No differences were observed in the total sensory scores, however PCA 28d rabbits were significantly less pain responsive [2 (2–3) vs. 3 (2–3); p = 0.006] and failed more frequently with the righting reflex [4 (4–5) vs. 5 (5–5); p < 0.019] (Fig. 1).

### Preterm rabbits have lower neuron densities in all brain regions assessed

**Figure 2:**
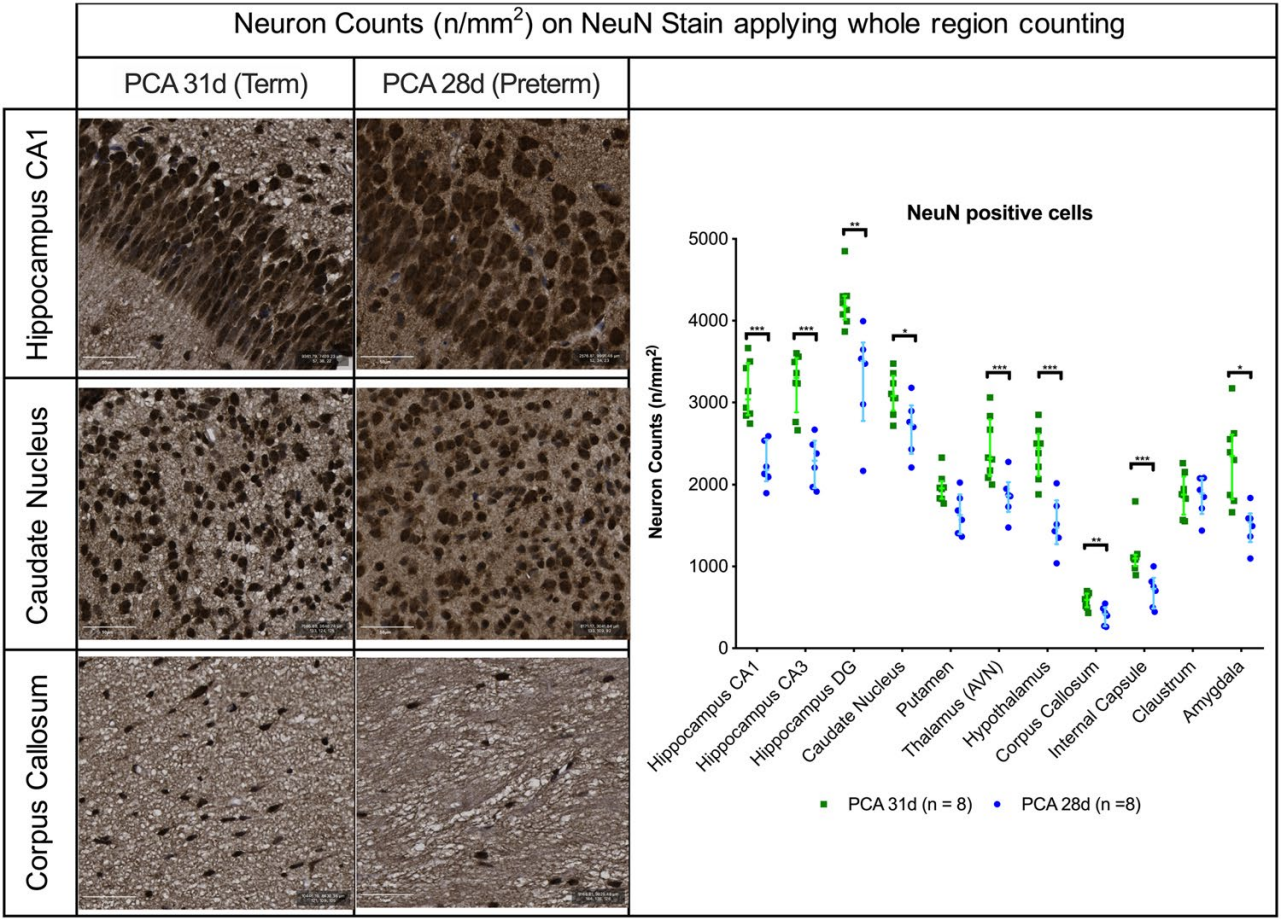
Neuron quantification with NeuN staining on PCA 31d in selected regions of interest. Representative images from hippocampus, caudate nucleus and corpus callosum with scale bar 50 µm. PCA 28d n = 8, PCA 31d n = 8. Data displayed as median and IQR with significance as *0.05 ≥ p > 0.01; **0.01 ≥ p > 0.001; ***p < 0.001.

Preterm birth led to significant lower neuron counts overall. On Nissl staining, with semi-automated whole region counting, there were significantly less neurons in all regions (Supplementary Fig. 5). Neuron specific staining confirmed the significantly lower neuron densities in all the regions except for the putamen [1628 (1397–2027) vs. 1956 (1829–2044) neurons/mm^2^; p = 0.023] and claustrum [1893 (1644–2080) vs. 1878 (1634–2259) neurons/mm^2^; p = 0.806] (Fig. 2). Of note, the proportion of cells not staining with NeuN immunohistochemistry varied between specific regions in the PCA 28d group but was as high as 7.7% in the corpus callosum (Supplementary Fig. 5).

### Lower neuron densities of preterm birth paralleled lower proportions of oligodendrocyte precursors

**Figure 3:**
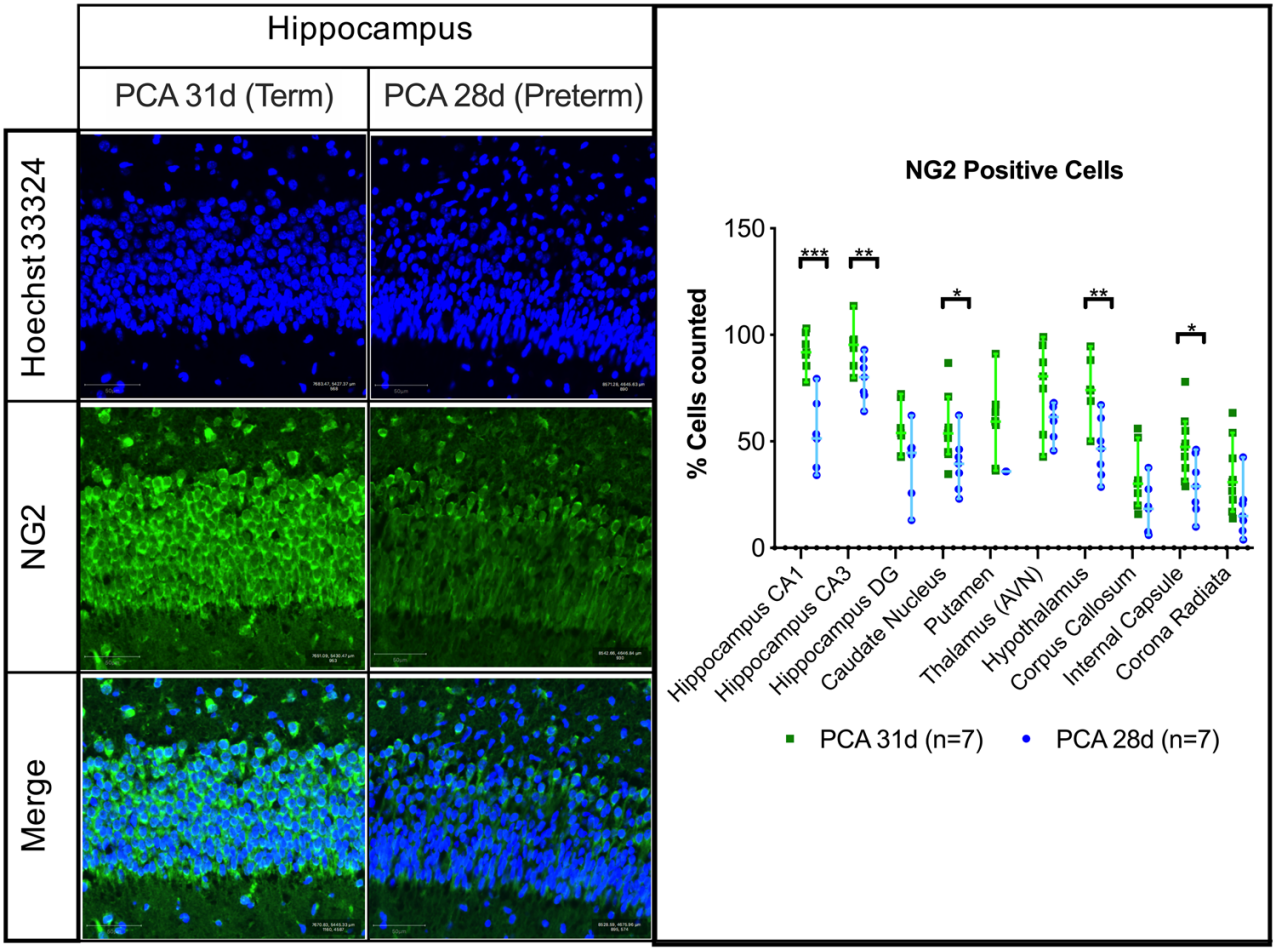
NG2 positive cells in selected regions of interest. Representative fields from the hippocampus CA1 region with scale bar 50 µm. PCA 28d n = 7, PCA 31d n = 7. Data displayed as median and IQR with significance as *0.05 ≥ p > 0.01; **0.01 ≥ p > 0.001; ***p < 0.001.

Less oligodendrocyte precursor cells were present in the PCA 28d group, as quantified by the number of NG2+ cells, in the hippocampus [51 (34–79) vs. 91 (77–100); p < 0.001], caudate nucleus [39 (23–62) vs. 53 (34–86); p = 0.036], hypothalamus [46 (28–67) vs. 74 (50–94); p = 0.002] and internal capsule [29 (9–46) vs. 47 (28–77); p = 0.025] (Fig. 3).

### Preterm brains have more pyknotic, apoptotic and astroglial cells and less proliferating cells

**Figure 4:**
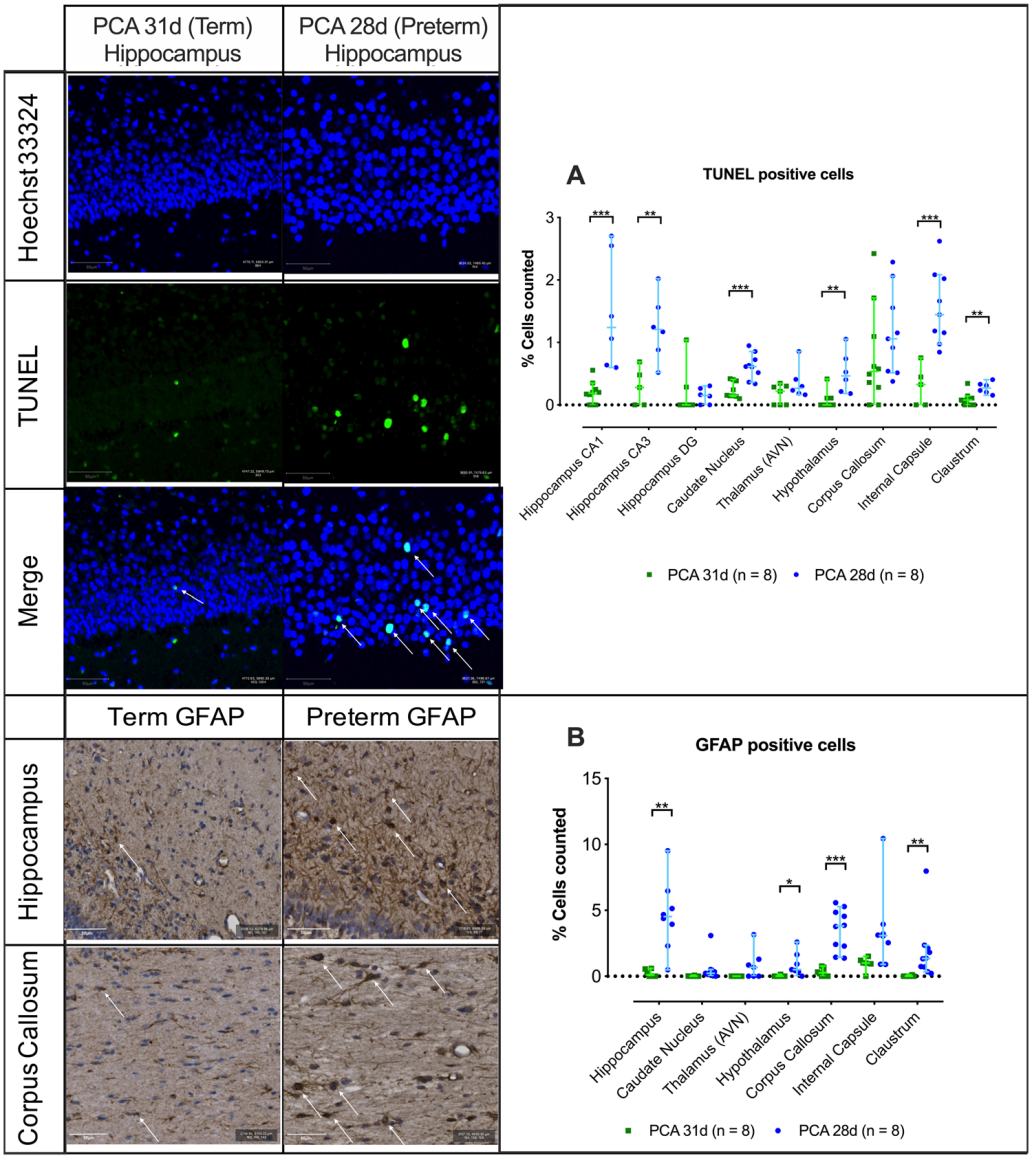
(**A**) TUNEL positive staining in selective regions of interest. Representative field from hippocampus CA1, scale bar 50 µm. (**B**) GFAP positive cell quantification in selected regions of interest with representative field from hippocampus and corpus callous, scale bar 50 µm. Data displayed as median and IQR with significance as *0.05 ≥ p > 0.01; **0.01 ≥ p > 0.001; ***p < 0.001.

In the preterm brains more pyknotic cells, with Cresyl Violet staining, were seen in the internal capsule [10.24% (1.55–30.92) vs. 4.92% (0.69–7.97); p = 0.048] and claustrum [5.43% (2.19–19.85) vs. 1.67% (0.47–3.17); p = 0.003] (Supplementary Fig. 5). Moreover, on direct apoptotic cell detection the proportion of TUNEL positive cells was higher in most of the PCA 28d brain regions; hippocampal region CA1 [1.24% (0.60–2.70) vs. 0.18% (0.0–0.56); p < 0.001] and CA3 [1.21% (0.52–2.02) vs. 0.28% (0.0–0.69); p = 0.006], the caudate nucleus [0.62% (0.34–0.95) vs. 0.15% (0.10–0.41); p < 0.001], hypothalamus [0.47% (0.18–1.05) vs. 0.01% (0.0–0.41); p = 0.005], internal capsule [1.45% (0.85–2.62) vs. 0.32% (0.0–0.75); p = 0.009] and claustrum [0.27% (0.15–0.41) vs. 0.08% (0.0–0.34); p = 0.003] (Fig. 4).

**Figure 5:**
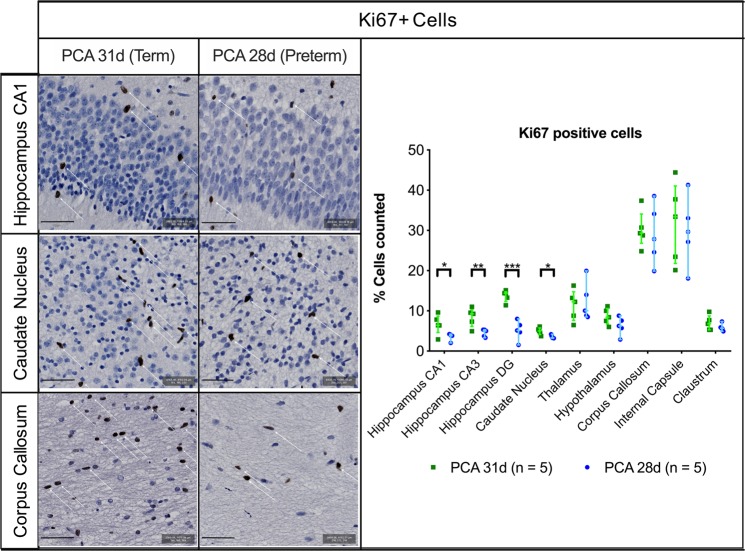
Ki67 positive cell quantification in selected regions of interest with representative images from hippocampus CA1, caudate nucleus and corpus callous, scale bar 50 µm. PCA 31d n = 5, PCA 28d n = 5. Data displayed as median and IQR with significance as * 0.05 ≥ p > 0.01; **0.01 ≥ p > 0.001; ***p < 0.001.

These differences were echoed in the higher proportion of astrocytes in the hippocampus [4.52% (0.49–9.51) vs. 0.11% (0.0–0.59); p = 0.001], hypothalamus [0.51% (0.01–2.60) vs. 0.02% (0.0–0.14); p = 0.035], corpus callosum [3.81% (1.38–5.58) vs. 0.26% (0.0–0.78); p < 0.001] and the claustrum [1.35% (0.22–7.97) vs. 0.00% (0.0–0.11); p = 0.016] of the PCA 28d group (Fig. 4). Additionally, significantly lower percentage of proliferating (Ki-67+) cells were noted in the hippocampus CA1 [3.82% (2.01–4.20) vs. 6.41% (2.89–9.59); p = 0.041], hippocampus CA3 [5.00% (3.30–5.30) vs. 9.25% (4.93–11.00); p = 0.004], dentate gyrus [5.12% (1.53–8.02) vs. 14.53% (11.38–15.11); p < 0.001] and caudate nucleus [3.29% (3.19–4.19) vs. 5.01% (3.70–6.10); p = 0.013] of the PCA 28d rabbits (Fig. 5).

### PTB resulted in altered synaptophysin expression in the hippocampus

The mean fluorescent intensity of synaptophysin, a major integral membrane glycoprotein of neuronal synaptic vesicles present in all synapses, was evaluated in the regions that noted the most significant changes and only a difference was noted in the hippocampus [716 (591–785)] vs. 872 (695–989); p = 0.02] (Supplementary Fig. 5).

### Region specific histologic changes coincided with MRI volumetric and diffusion weighted imaging

**Figure 6:**
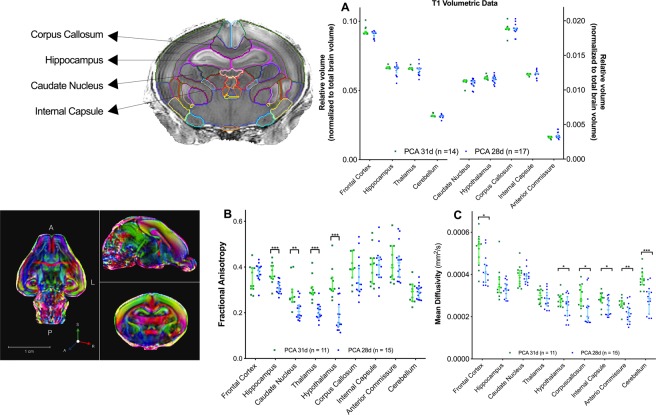
*Ex-vivo* MRI derived data per region of interest at PCA 32 days. (**A**) T1-weighted relative volumes normalised to total brain volume with representative image of segmented volume. (**B**) DTI fractional anisotropy and (**C**) DTI mean diffusivity data with representative image of DTI acquisition. Data displayed as median and IQR with significance as *0.05 ≥ p > 0.01; ** 0.01 ≥ p > 0.001; ***p < 0.001.

T1-derived volumetric data substantiated the overall region-specific histological findings with the PCA 28d rabbits having an overall lower absolute brain volume. When normalizing these volumes to their respective body weight, the PCA 28d rabbits had overall a relative larger volume per region of interest (Supplementary Fig. 6). The most significant differences were noted in the thalamus, hippocampus, corpus callosum, hypothalamus and internal capsule (all p < 0,001). Yet, when the proportionality of these brains is evaluated, normalizing the regional volumes to their respective total brain volume, no proportional differences were present between the two groups (Fig. 6).

Differences on DTI also paralleled the histological findings. Fractional anisotropy (FA) was significantly lower in the hippocampus (0.31 ± 0.025 vs. 0.37 ± 0.045; p < 0.001), caudate nucleus (0.21 ± 0.041 vs. 0.29 ± 0.061; p = 0.001), thalamus (0.20 ± 0.033 vs. 0.31 ± 0.050; p < 0.001) and hypothalamus (0.18 ± 0.062 vs. 0.33 ± 0.067; p < 0.001) of the PCA 28d rabbits. Mean diffusity (MD) was lower in the corpus callosum (0.232 × 10^−3^ mm^2^/s ± 0.070 vs. 0.292 × 10^−3^ mm^2^/s ± 0.051; p = 0.023), internal capsule (0.243 × 10^−3^ mm^2^/s ± 0.039 vs. 0.282 × 10^−3^ mm^2^/s ± 0.033; p = 0.011), anterior commissure (0.215 × 10^−3^ mm^2^/s ± 0.045 vs. 0.260 × 10^−3^ mm^2^/s ± 0.029; p = 0.005) and cerebellum (0.281 × 10^−3^ mm^2^/s ± 0.062 vs. 0.378 × 10^−3^ mm^2^/s ± 0.044; p < 0.001) (Fig. 6).

Of the planned 40 acquisitions a total of 35 (16 term 19 preterm) were done. Four of the PCA 31d subjects (due to artefacts from the perfusion fixation, diffusion imaging artefacts and ghosting artefacts) and three of the PCA 28d (all due to motion artifacts) acquisitions were excluded. An interim power calculation, based on the FA data in the hippocampus, confirmed a power of 95% to detect these differences. Particulars of the subjects that underwent MRI acquisitions are in Supplementary Fig. 6.

### The role of sex in this model

When exploring whether the sex of the rabbit related to the above outcomes, no overall association was noted. Yet, isolated findings were noted in the male sex. PCA 28d male rabbits had a higher hippocampal FA (0.33 ± 0.020 vs. 0.29 ± 0.013; p = 0.001) and PCA 31d male rabbits were associated with a higher neuron density in the caudate nucleus [3408/mm^2^ (3064–3653) vs. 2943/mm^2^ (2543–3123); p = 0.011]. (Supplementary Fig. 7).

## Discussion

This small animal model elicits the effect of prematurity in the absence of any contributing infective or hypoxic-ischemic insult. The multimodal assessment approach enabled the correlation of distinct neurobehavioral deficits with neuropathological and neuroimaging changes. The premature newborns, at corrected equivalent age, had a clear motoric and partial sensory deficit, overall lower neuron densities, that was accompanied by reduced oligodendrocytes wherein more apoptosis and less proliferation was noted. Additionally, these differences coincided with MRI-derived DTI deviations in key regions that are typically associated with EoP, i.e. the hippocampus, caudate nucleus, thalamus^28^.

A recent review specified criteria that preclinical models of EoP should fulfil to best aid current translational objectives^29^. This model fulfils all criteria as it produced a spectrum of functional deficits, addressed the multiple components of injury evident throughout the CNS and included similar mechanisms of prenatal global injury observed in EoP. The effect of prematurity was seen in the absence of proven contributors like hypoxia-ischemia, infection or even intraventricular hemorrhage. Making it more relevant to the common prematurity setting. In view of the high mortality, severe morbidity and dependence upon additional support for survival, these preterm rabbits mimic the clinical scenario of extreme prematurity^30^. To define precise gestational age equivalents between humans and any animal is practically impossible since organs can have species specific independent developmental trajectories. The rabbit gestation of 28 days shares some characteristics of a 20–28 week human gestation based upon their altricial status and the stage of their brain^6^ and lung development^31^.

Counterintuitively, the gestational equivalent of 31 days in rabbits is even harder to define. While these newly born rabbits can independently suckle, maintain their homeostasis and have strong enough locomotor capabilities to explore their nest, their brain^6^ and lung development^31^ are still more in keeping with early prematurity in humans.

More relevant than direct structural comparisons is the biological significance of neurogenesis in this model. Rabbit CNS development is protracted for long periods into postnatal life before attaining tissue maturation (e.g. in the striatum and cerebellum)^32^. In this context, rabbits are ideal models for studying structural plasticity and neurogenesis, since this may represent a potential interventional window.

The utility of the early neurobehavioral assessment to detect a functional deficit was confirmed, as noted in other rabbit models that studied the perinatal effect of hypoxia-ischemia^17^, intraventricular hemorrhage^20^ and intrauterine growth restriction^33^. These motor deficits form a major part of the clinical context of EoP^5^ and it is imperative for any preclinical model to correlate functional neurological outcomes to the underlying neuropathology processes if any mechanism will be investigated to modulate the insult.

Beyond this motoric insult, it is essential to explore other early neurobehavioral assessment methods to predict medium- to long-term neurological outcomes. Early ethological and vocalization behaviors of the neonatal rabbit^34^ can potentially add towards a more comprehensive neurobehavioral assessment that will not be dominated by the motoric insult. According to what current clinical research is attempting^35^, if the clinical utility of an early assessment method could be proven this could highlight a subgroup that requires further characterization to find prospective targets for manipulation.

While the purpose of this study was to construct a more appropriate model of EoP, some mechanistic processes already became apparent. The motoric deficit was noted in context of distinctive changes in the hippocampus and thalamus wherein reduced neuron density was accompanied by less proliferation and increased inflammation. Similar to what has been described in human perinatal brain injuries^36^, it seems that premature rabbits have an intrinsic vulnerability to neuronal damage. To further explore this, the role of NG2 glial cells was studied. Neural progenitor cells expressing chondroitin sulphate proteoglycan 4, known as NG2 glial cells (or oligodendrocyte progenitor cells), migrate from the germinal zones, actively proliferate, and differentiate into oligodendrocytes that form myelinated tracts during early postnatal life. NG2 glial cells comprise the majority of the proliferative cells in the CNS and can rapidly balance proliferation and migration to restore their density in response to focal cellular loss^37^. We confirmed that in the hippocampus NG2 glial cells were significantly reduced and this supported the hypothesis that NG2 glial cells maintain the neural environment under normal physiological conditions and that the dysfunction of these cells, as in the preterm rabbits, leads to impairment of neuronal function.

The above mentioned neuropathological changes also coincided with MRI data alterations of the regions involved in the corticothalamic tracts. The DTI differences in these formative motoric pathways are most likely explained by their maturational vulnerability leading to reduced extra-axonal space and therefore less packing of fibers^38^. Yet, it could also be due to cytotoxic edema mediated by an inflammatory mechanism^38,39^. This is supported by the finding of more astroglia cells in preterm rabbits.

Additionally the abnormal white matter maturation, which is seen in premature infants^40^, follows the same hierarchy of anisotropy differences as in the clinical scenario^41^. The FA first appears to increase in the commissural tract and corpus callosum. Hence, suggesting similar maturational patterns in deep white matter tracts. Moreover, these differences where not influenced by the PCA as both groups where of similar chronological ages. This is once again similar to findings in term-equivalent preterm neonates, wherein persisting microstructural changes in WM pathways are noted, even when correcting for the chronological age of the child^42^.

The strengths of this model rest in the multimodal assessment approach and the multiregional analyses in view of the fact that the nature of the prematurity insult incorporates a multiregional multicomponent effect^28^. Additionally, the role of sex differences, as observed in human clinical studies, has been considered in this model^43^. Lastly, the utility of animal models is best illustrated by the fact that underlying cellular changes and mechanisms can be evaluated, thereby aiding the critical evaluation of underlying pathology and in the development of therapeutic interventions.

As no animal model can perfectly mimic the human condition, the present translational research has some limitations. This small animal model cannot replicate the exact scenario of prematurity in humans since precise human GA equivalents are practically impossible. Another concern is the impact of neonatal care for the rabbits and although optimal conditions and feeding regimes are adhered to, this variable can account for many alterations, as in humans. Moreover, the mortality seen with premature delivery cannot be easily addressed since causation cannot be established and preemptive management might add another confounder to this study. When reflecting on the outcomes, the early neurobehavioral assessment is limited to noting motoric differences and no clear cognitive impact can be elicited at this time point. Also the phenotypic variation following early CNS injury can represent an integral weakness. Likewise, there is some overlap within the neuropathological and DTI data that stresses the heterogeneity of model. Although this variation also occurs in humans, it should be considered when the efficacy of novel interventions is evaluated as it could impact the variation of severity, recovery, repair, and treatment response. Lastly, since rabbits are perinatal brain developers with post-natal myelinization, no evaluation could yet be made about possible impaired or delayed myelinization, a key feature of EoP and exemplified in many animal models of ischemic EoP^44^.

In conclusion, in this small animal multimodal assessment model, prematurity in the absence of any other contributory events resulted in clear neurobehavioral changes that correlated with histopathological and MRI findings. This rabbit model echoes the key features of EoP and enables the possibility to evaluate various important antenatal insults.

## Materials and Methods

### Animal delivery and care

Animals were treated according to current guidelines for animal well-being, and all experiments were approved by the Ethics Committee for Animal Experimentation of the Faculty of Medicine (P062/2016). Time-mated pregnant rabbit does (Oryctolagus cuniculus; Hybrid of Dendermonde and New Zealand White) were housed in separate cages before delivery, with free access to water and chow and a light-dark cycle of 12 h. The does underwent a caesarean delivery at a postconceptional age (PCA) of either 28 (pre-term, n = 14) or 31 (term, n = 12) days. The New Zealand white rabbit has a gestation period of 31 days and delivery before PCA 28 invariably results in death (data not shown here)^24^.

Before delivery, rabbits were weighed and premedicated with intramuscular ketamine (15 mg/kg, Nimatek®; Eurovet Animal Health BV, Bladel, The Netherlands) and medetomidine (25 mg/kg, Domintor®, Orion Pharma, Aartselaar, Belgium). The doe was placed in supine position and local anesthesia (2% lidocaine hydrochloride, Xylocaine®, AstraZeneca, Brussel, Belgium) was injected before the skin incision. Following delivery, the doe was euthanized with a mixture of 200 mg embutramide, 50 mg mebezonium, and 5 mg tetracain hydrochloride (intravenous bolus of 1 mL T61®; Intervet International BV, Boxmeer, The Netherlands).

At delivery, the kittens were dried, stimulated, weighed and placed in an incubator (TLC-50 Advance, Brinsea® Products, Weston Super Mare, UK) at 32 °C with 60% humidity as described^45^. Kittens were fed twice per day, via a 2.5 Fr orogastric tube, with a milk replacer containing 30% proteins and 50% of lipids (FoxValley 30/50, Lakemoor Illinois, US), Bio-Lapis for electrolytes, vitamins and probiotics (Protexin Veterinary, Somerset, UK) and Col-o-cat for a high amount of immunoglobulins (Sanobest, ‘s Hertogenbosch, Netherlands). The feeding increased from 75 to 100 to 150 mL/kg/feeding on PN days 0, 1, and 4 respectively. The kittens remained in the incubator except during feeding.

Both groups, PCA 28d (preterm) and PCA 31d (term), were evaluated at PCA of 32 days. Kittens were randomly selected for either histopathological or MRI analysis with Research Randomizer (http://www.randomizer.org). To determine whether animals displayed adverse physiological consequences that might interfere with further testing, heart rate and rectal temperature was measured in randomly selected newborns yet no animals were excluded.

### Neurobehavioral testing

Neurobehavioral evaluations were carried out early mornings before feeding using a modified neurobehavioral scoring protocol described previously^16,19^. Done in a designated space close to the incubator with auditory and olfactory contamination kept to a minimum. The motor function (tone, motor activity, and locomotion, righting reflex, and gait) and sensation (touching the whiskers with cotton swab and a mild pin prick to evaluate pain sensation on the hind limbs) including cranial nerves (olfaction, sucking and swallowing and head turn to feeding) were assessed (Supplement 1). Evaluations were videotaped and scored by two observers blinded to the group allocation. Inter- and intraobserver reliability for the neurobehavioral scoring during the pilot study were assessed (Supplementary Fig. 2).

### Harvesting and tissue collection

Neonatal rabbits were anesthetized with intramuscular ketamine (35 mg/kg, Nimatek®; Eurovet Animal Health BV, Bladel, The Netherlands) and xylazin (6 mg/kg, XYL-M®; VMD, Arendonk, Belgium) and transcardially perfused with 0.9% saline and heparin (100 u/mL) followed by perfusion fixation with 4% paraformaldehyde (PFA) in 0.1 mol/L phosphate buffer (pH 7.4). After extraction the brain was further immerse fixated for 48 h and then paraffin embedded. Whole brain volumes, including cerebellum, were determined using a fluid displacement method^46,47^.

### Histopathology

Brains were microtomed at 4 µm thickness from anterior to posterior. A set of 5 serial coronal sections at 100 µm intervals was taken at each of the following two levels. The first level started at the medial septal nucleus and second at the hippocampal formation. Supplementary Fig. 3 illustrates the regions of interest. Sections were placed onto poly-L- lysine coated slides (Sigma-Aldrich, Bornem, Belgium). Staining was done with Cresyl Violet (C5042-10G, Sigma-Aldrich, Overijse, Belgium) and the primary antibodies used included mouse monoclonal anti-NeuN antibody (MAB377, Millipore, Billerica, MA, USA), anti-NG2 chondroitin sulfate proteoglycan antibody (MAB5384, Millipore, Billerica, MA, USA), mouse monoclonal anti-glial fibrillary acidic protein antibody (GFAP) (G6171, Sigma-Aldrich, St Louis, MO, USA), mouse monoclonal anti-human Ki67 (M724001-2, Agilent, Diegem, Belgium), mouse monoclonal anti-synaptophysin (Sy38, ab8049, Abcam, Cambridge, UK). The secondary antibody was Alexa Fluor® 488 goat anti-mouse conjugate (Invitrogen). Degenerating nuclei were visualized by a terminal deoxynucleotidyl transferase dUTP nick end labelling (TUNEL) method for fluorescent *in situ* end labelling of double-stranded DNA fragmentation (Apoptag S7110; Millipore, Billerica, MA, USA). Sections were counterstained with Hoechst 33342 (Sigma-Aldrich, Bornem, Belgium).

### Imaging acquisition and quantification

Histological slides were digitized using the Zeiss AxioScan Z1 imaging platform (AxioScan® Slide Scanner, Carl Zeiss MicroImaging GmbH, Munich, Germany), using a 20 x Plan Apochromat objective coupled to a 3 Chip CCD Camera (Hamamatsu Photonics, Japan). Focusing and field of view assembly was handled by the Carl Zeiss Zen software (Carl Zeiss MicroImaging GmbH), integrated with the AxioScan device. Quantification profiles of the digitized whole slide images were done using QuPath software^48^ under either bright field fast cell counting, or fluorescent positive cell detection settings. To differentiate between cellular types in each stain the detection classifier function^49^ was used with nucleus detection settings.

*Neuronal densities* were expressed as the average number per surface area per animal. Digital whole slide Nissl stained images were quantified with the CellProfiler function^49^ of the QuPath software to differentiate between neuronal, pyknotic and other cells based upon the morphological criteria for neurons and the optical density. Cresyl violet stained neurons were defined as cells having diameters >5 µm and containing a dark nucleolus within a lightly stained nucleus and a more heterogenous cytoplasm i.e. Nissl substance^50,51^.

*Quantification of the immunohistochemistry positive cells* with the NeuN, TUNEL, GFAP and Ki67 stains was done with the positive cell detection function as stated. Herein whole region of interest was annotated, quantified and positive cell counts were expressed as percentage of positive cells per total cells detected. For the mean fluorescence intensity quantification per region of interest of the synaptophysin stain (Sy38) open source Fiji software (ImageJ) (http://fiji.sc/Fiji) was used.

### MRI

*Ex vivo MRI* was performed on perfused fixed brains using the active staining technique as described^27,52^. Briefly, a Bruker Biospec 9.4 Tesla small animal MR scanner (Bruker Biospin, Ettlingen, Germany; horizontal bore, 20 cm) equipped with actively shielded gradients (600 mT/m) was used. Data was acquired using a 72 mm internal diameter quadrature volume coil for transmission decoupled with a rat brain quadrature shaped surface coil for signal reception (volume resonator, Rapid Biomedical, Rimpar, Germany). Data was acquired with a high-resolution 3D Flash sequence (TE/TR 5.5/50 ms; flip angle 70 degrees; slice thickness 0.35 mm with no interslice gap, data matrix 392 × 392 × 392; isotropic resolution 89μm; 4 averages, acquisition time 1h11min) and a SE-EPI sequence (8 segments; TE/TR: 25/150 ms; slice thickness 0.4 mm with no interslice gap, data matrix 192 × 160 × 160; isotropic spatial resolution of 208 μm; 64 directions and 3 b-values per direction of 800, 1000, 1500 s/mm^2^, acquisition time 10h26min).

Analysis was done with automated template propagation for the neonatal rabbit brain^27^ and subsequent T1 volumetric data and DTI derived fractional anisotropy (FA) and mean diffusivity (MD) were calculated. Data from the left and right hemispheres were summed together.

### SRY-gene detection

Molecular PCR (polymerase chain reaction) technique for the detection of SRY (specific region of Y chromosome) sequences was used to detect the male sex^53^. Fresh skin tissue was collected at harvesting for DNA isolation using the GenUP™ gDNA Kit (Biotechrabbit, Henningsdorf, Germany). About 200 ng DNA were used for PCR. Amplification of SRY fragment was done in 35 cycles under the following conditions: first denaturation step 94 °C 2 min then 94 °C 20 sec, 64 °C 30 sec, 72 °C 30 sec with last step 72 °C 10 min using specific primers OcSRY21F: 5′-AGC GGC CAG GAA CGG GTC AAG-3′, and OcSRY23R: 5′-CCT TCC GGC GAG GTC TGT ACT TG-3′. Obtained PCR products were analyzed by electrophoresis in 3% agarose gel containing ethidium bromide and visualized by GENESYS software package (version 1.2.0.0). In all samples rabbit GAPDH, primers GAPDHF: 5′-TCA CCA TCT TCC AGG AGC GA-3′, and GAPDHR: 5′-CAC AAT GCC GAA GTG GTC GT-3′ (487 bp), were used as an internal amplification control.

### Statistics

Preterm rabbits were expected to have a decreased neural density and lower FA on DTI. A priori power calculation was constructed using the neuron counts and MRI data from an initial pilot study using G*Power^54^. Although 10 rabbits per group were required to show a 5% difference (α = 0.05 and 1 - β = 0.80) in hippocampal neural densities, an adjustment based was made to power the study to also investigate a 5% difference in FA, eventually requiring 20 rabbits per group.

Data was analyzed using Prism for Windows version 5.0 (Graphpad software, San Diego, CA, USA). Data was checked for normality of distribution using a D’Agostino-Pearson omnibus normality test, then presented as a mean with standard deviations or median and interquartile ranges. Comparison was done by unpaired students t-test or Mann Whitney test. A p-value < 0.05 was considered significant. Adjustment for multiple testing was applied using the Benjamini–Hochberg false discovery rate set at 1% when more than 5 comparisons where done. A Grubbs’ test with an α of 0.05 was used to identify outliers. Survival curves are presented as a Kaplan-Meier graph with groups comparisons using a Mantel-Cox test. While Bland-Altman plots were used to assess inter- and intraobserver agreement.

## Supplementary information

Supplementary material
