# Publisher Correction: Early neuropathological and neurobehavioral consequences of preterm birth in a rabbit model

**DOI:** 10.1038/s41598-019-53779-x

**Published:** 2019-11-13

**Authors:** Johannes van der Merwe, Lennart van der Veeken, Sebastiano Ferraris, Willy Gsell, Uwe Himmelreich, Jaan Toelen, Sebastien Ourselin, Andrew Melbourne, Tom Vercauteren, Jan Deprest

**Affiliations:** 1 Department of Development and Regeneration, Cluster Woman and Child, Group Biomedical Sciences, KU Leuven University of Leuven, Leuven, Belgium; 2 Department of Obstetrics and Gynaecology, Division Woman and Child, University Hospitals Leuven, Leuven, Belgium; 3 Translational Imaging Group, Centre for Medical Image Computing (CMIC), Department of Medical Physics and Biomedical Engineering, University College London, London, UK; 4 moSAIC facility, Biomedical MRI, Department of Imaging and Pathology, KU Leuven, Leuven, Belgium; 5 Department of Pediatrics, Division Woman and Child, University Hospitals Leuven, Leuven, Belgium; 6 School of Biomedical Engineering and Imaging Sciences, King’s College London, London, UK; 7 Institute for Women’s Health, University College London, London, UK

Correction to: *Scientific Reports* 10.1038/s41598-019-39922-8, published online 05 March 2019

In the original version of this Article, the ORCID IDs for the authors Johannes van der Merwe, Lennart van der Veeken, Sebastiano Ferraris, Jaan Toelen and Jan Deprest were omitted. This has now been corrected in the HTML and PDF versions of the Article.

