# Supplementary material for: Early neuropathological and neurobehavioral consequences of preterm birth in a rabbit model
Source: Sci Rep. 2019 Mar 5;9:3506. doi: 10.1038/s41598-019-39922-8 (PMC6401068; doi:10.1038/s41598-019-39922-8)
Supplement: Supplementary file 1 — Supplementary material [file 41598_2019_39922_MOESM1_ESM.pdf]

Early neuropathological and neurobehavioral consequences of preterm birth in a rabbit model

Johannes van der Merwe[1,2], Lennart van der Veeken[1,2], Sebastiano Ferraris[3], Willy Gsell[4], Uwe Himmelreich[4], Jaan Toelen[1,5], Sebastien Ourselin[3,6], Andrew Melbourne[3], Tom Vercauteren[1,3,6], Jan Deprest[1,2,7]

[1]Department of Development and Regeneration, Cluster Woman and Child, Group Biomedical Sciences, KU Leuven University of Leuven, Belgium

[2] Department of Obstetrics and Gynaecology, Division Woman and Child, University Hospitals Leuven, Leuven, Belgium

[3]Translational Imaging Group, Centre for Medical Image Computing (CMIC), Department of Medical Physics and Biomedical Engineering, University College London, London, UK

[4]moSAIC facility, Biomedical MRI, Department of Imaging and Pathology, KU Leuven, Belgium

[5]Department of Pediatrics, Division Woman and Child, University Hospitals Leuven, Leuven, Belgium

[6]School of Biomedical Engineering and Imaging Sciences, King's College London, London, UK

[7]Institute for Women's Health, University College London, London, UK

Rabbit kit: Early neurobehavioral assessment PN+1d		Date of evaluation: |__|__| |__|__| |__|__|	
**Rabbit ID:** |__|__|__|__|	**Weight (g):**	**PN day: +**	**Video ID:**
During adaptation: |__| Sleepy |__| Awake Some activity |__| Active and exploring			

NEUROMOTORIC

**A. Posture**	
**0**	Lays supine
**1**	Lays on side/minimal movement
**2**	Cannot maintain prone position (roll over > 50% time)
**3**	Prone position with legs coiled (roll over < 50% time)

**B. Gait** (2)	
**0**	Not able to move/uses whole body to move
**1**	Crawls, trunk touching ground, unstable > 50% time
**2**	Walks, trunk low/touching ground, unstable <50% time
**3**	Walks but can't propel body using hind legs synchronously
**4**	Walks, runs, and hops (synchronous) and coordinated

**C. Locomotion**(4) [all criteria must be met]	
**0**	Not able to move/uses whole body to move
**1**	Some movement, single steps, slight hops, <15cm
**2**	Good ROM, 2-3 continuous steps, 1-2 hops, 15-45cm
**3**	Entire ROM, >3 continuous steps, >3 hops, >45cm
	Maximum number of continuous steps
	Total distance travelled (cm)
	Total number of squares crossed (n)

**D. Motor Activity**	Head	Fore limbs	Hind limbs
**0** No Movement			
**1** Slight Movement			
**2** Distinct Movement			
**3** Rapid/Purposeful Movement			

**E. Duration of activity during observation**	
**0**	No movement
**1**	Active for < 20 seconds
**2**	Active for 20-40 seconds
**3**	Active for > 40 seconds

**F. Limb Tone (Hind limbs)** (5)	
**0**	Limb rigid in flexion or extension
**1**	Tone considerably increased, passive movement difficult
**2**	Marked increase in tone but limb is easily flexed
**3**	Slight increase in tone when limb is moved
**4**	No increase in tone

NEUROSENSORY

**G. Surface Righting Reflex (5 attempts)** (2)	
	no. of times turned to prone position within 2 sec seconds when placed in supine position

**H. Sensation - Facial/Whisker touch response**	
**0**	No Response
**1**	Subtle (none directed reaction after ≥ 3 stimuli)
**2**	Mild (slow or directed response after 2 stimuli)
**3**	Immediate avoidance or brushes side of head

**I. Sucking and swallowing**	
**0**	No movement of jaw, milks dribbles out completely
**1**	Some jaw/neck movement, most milk dribbles out
**2**	Definite suck and swallow, some milk in nose
**3**	Good suck and swallow, no milk in nose

**J. Head turning during feeding**	
**0**	No movement
**1**	Slow, occasional movement of head
**2**	Distinct movement of only head
**3**	Rapid forceful movements of head and body

**K. Odour Aversion Test**		Ethanol	
**0**	No Response (>20s)		
**1**	Subtle (6-20se)		
**2**	Low (2-5sec)		
**3**	Rapid/Normal (<2sec)		

**L. Pain sensation – response to pin prick on limbs**	
**0**	No Response within 20 seconds with >3 pricks
**1**	Subtle (>2seconds, response with >2 pricks)
**2**	Mild (<2 seconds, rapid response with >1prick)
**3**	Rapid/immediate, repetitive movement

# Performed on designated 25 x 25 cm area divided into 5 x 5 cm blocks over a time period of 90sec.

Supplement 1. Neurobehaviuoral assessment protocol

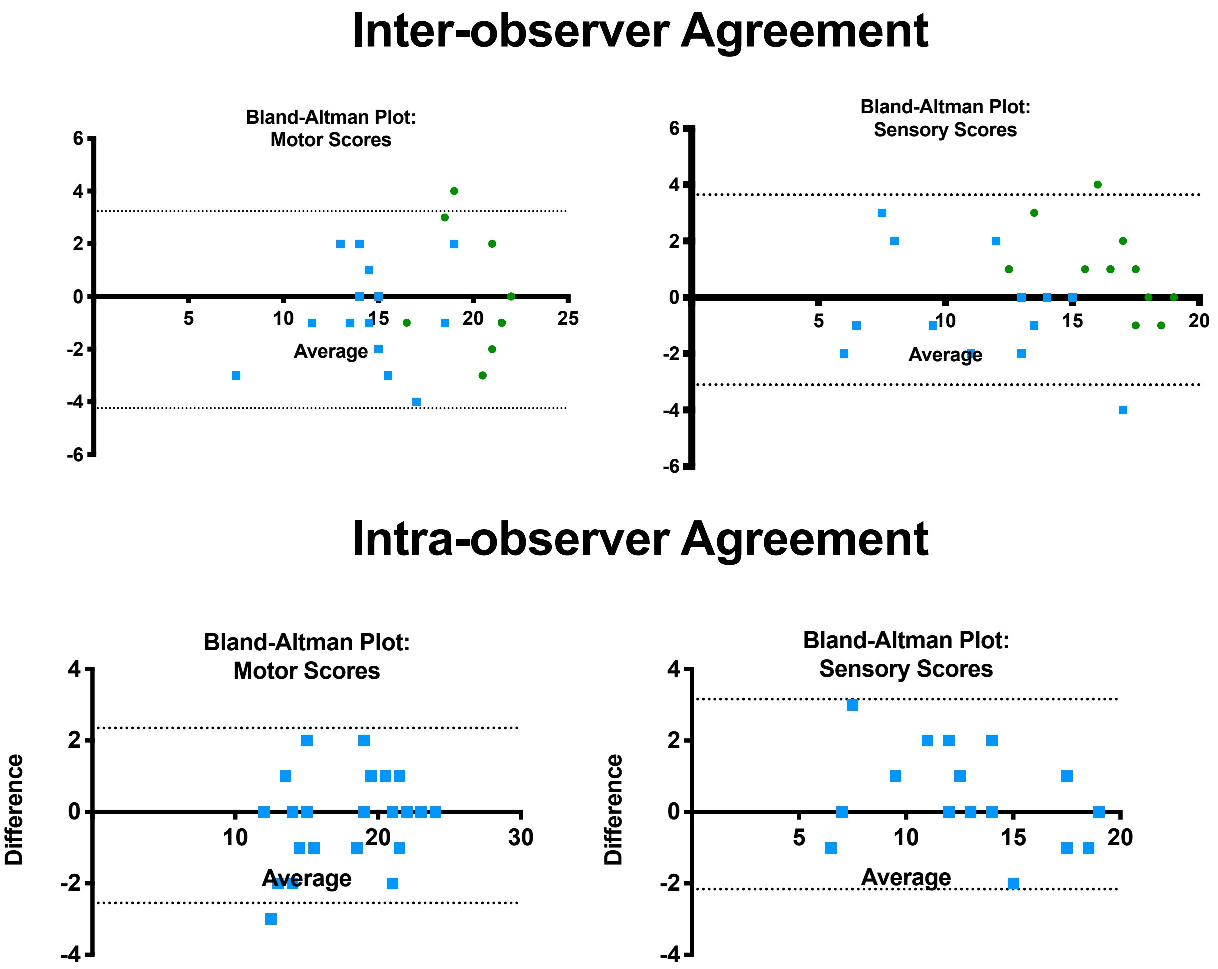

Supplementary Figure 2: Bland Altman plot for the intra- and interobserver agreement in neuromotor and sensory scores. To test for the reliability of the neurobehavioral assessment, 30 randomly selected neurobehavioral videos were analyzed by 2 observers.

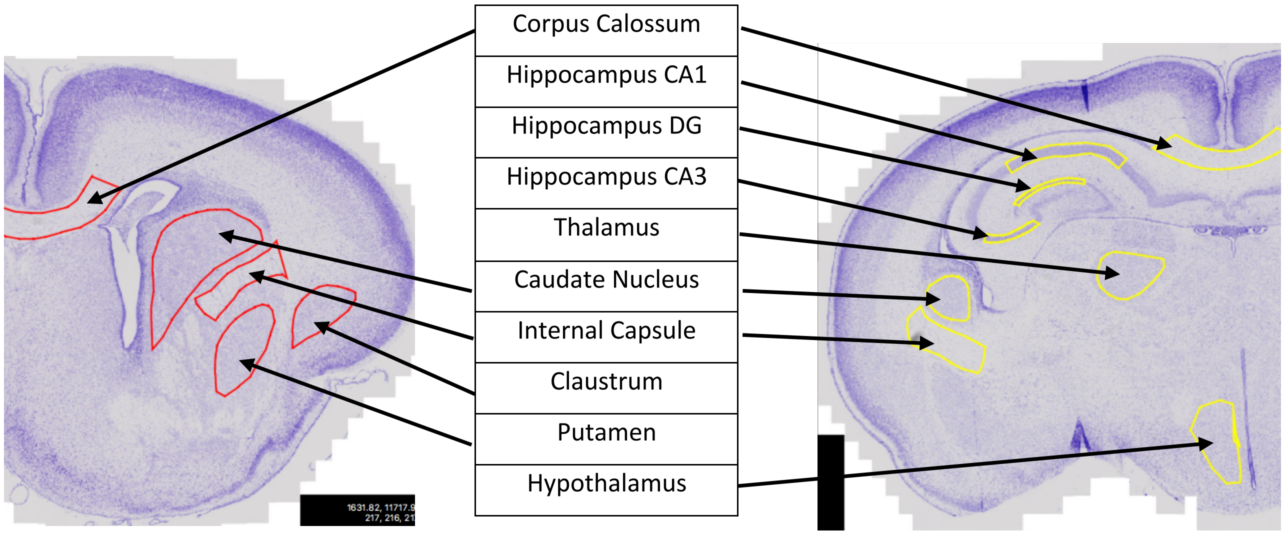

Supplementary Figure 3. Regions of interest annotated on the two levels taken serially from each brain. From the first set of coronal sections, the corpus callosum, caudate nucleus, putamen, internal capsule and claustrum were annotated. On the second set the corpus callosum, hippocampal regions CA1, CA3 and DG, caudate nucleus, the anterior ventral nucleus of the thalamus, internal capsule, claustrum and hypothalamus.

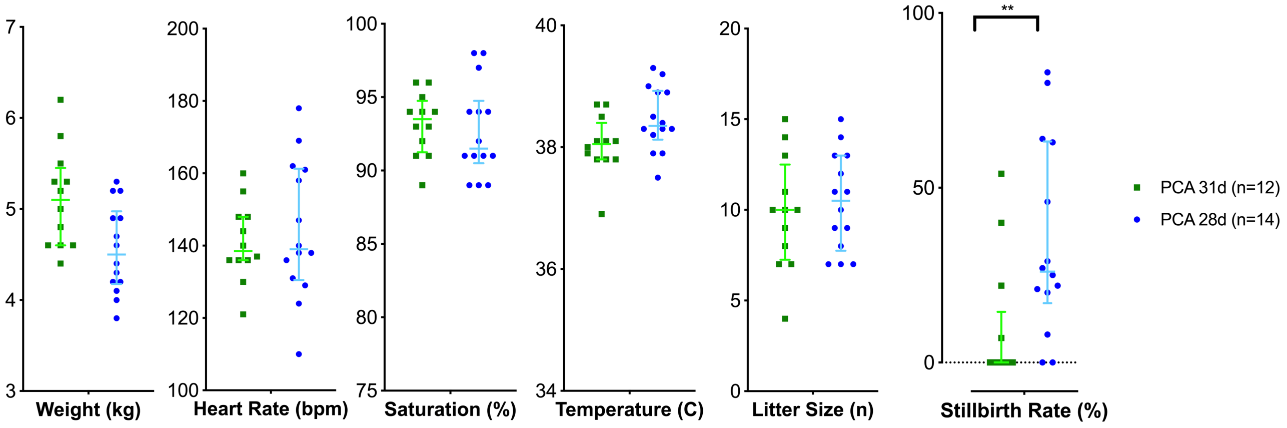

Figure 4a. Maternal characteristics of pregnant rabbit does at the time of delivery. PCA 31d n=12, PCA 28d n=14. Data displayed as median and IQR with significance as * 0.05 ≥ p > 0.01; ** 0.01 ≥ p > 0.001; ***p < 0.001.

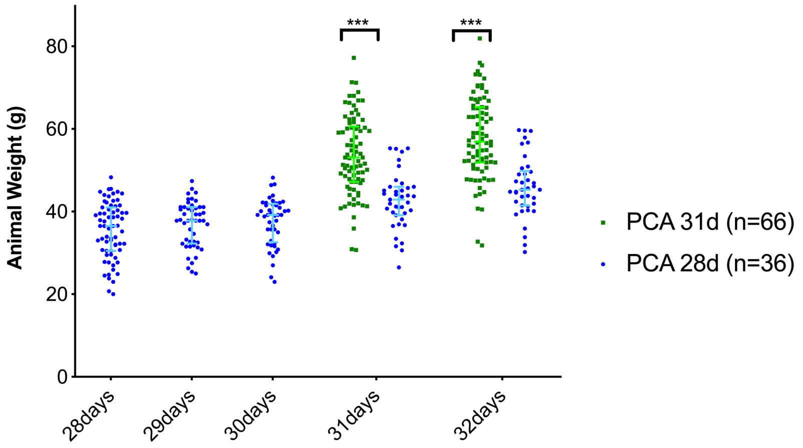

Figure 4b. Postnatal weight of newborn rabbits. PCA 31d n=66, PCA 28d n=36. Data displayed as median and IQR with significance as * 0.05 ≥ p > 0.01; ** 0.01 ≥ p > 0.001; ***p < 0.001.

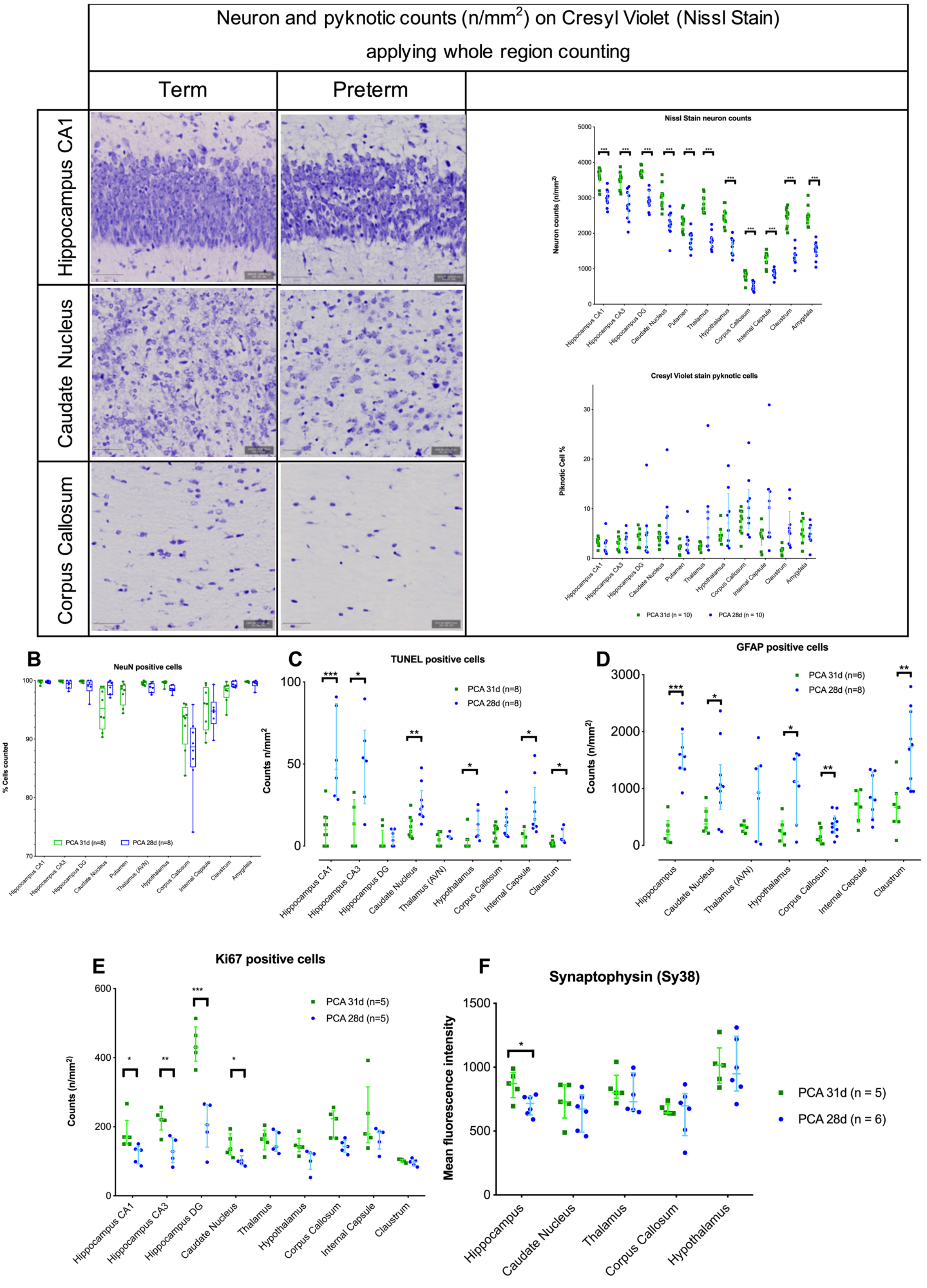

Figure 5. (A) Neuron and pyknotic cell counts (n/mm2) on Cresyl Violet (Nissl Stain) applying whole region counting. Representative fields from main regions of interest. Scale bar 50μm. **(B)** % NeuN positive cells in each region of interest. **(C,D,E)** Total number of positive cells for TUNEL, GFAP and Ki67 stains per region of interest. **(F)** Sy38 positive cells mean fluorescence intensity in selected region of interest. Data displayed as median and IQR with significance as * 0.05 ≥ p > 0.01; ** 0.01 ≥ p > 0.001; ***p < 0.001.

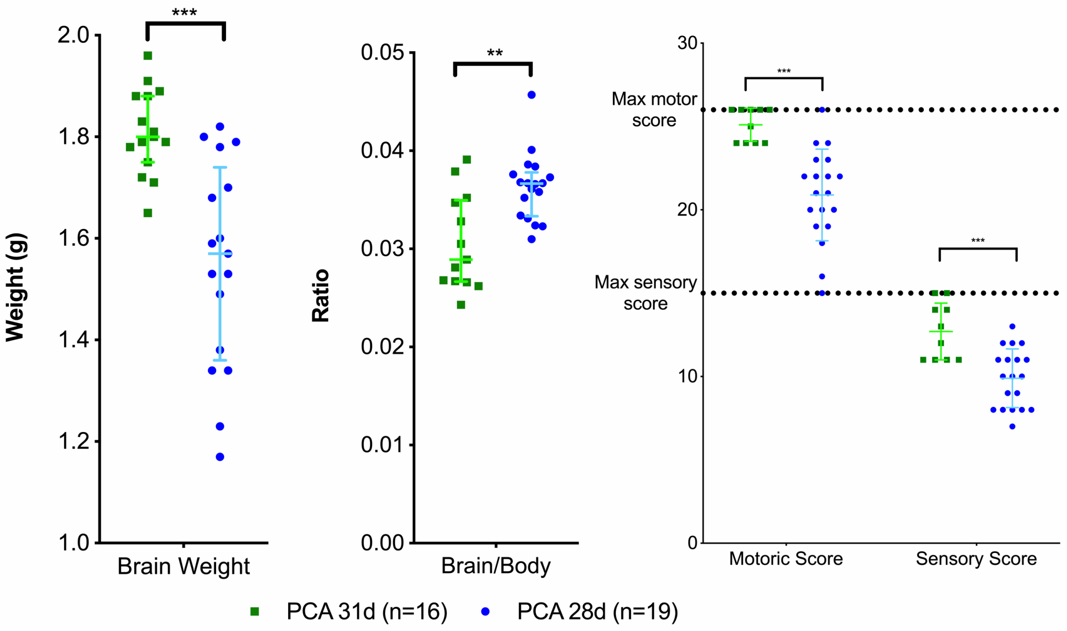

Figure 6a. Characteristics of the subjects that underwent MRI. Data displayed as median and IQR with significance as * 0.05 ≥ p > 0.01; ** 0.01 ≥ p > 0.001; ***p < 0.001.

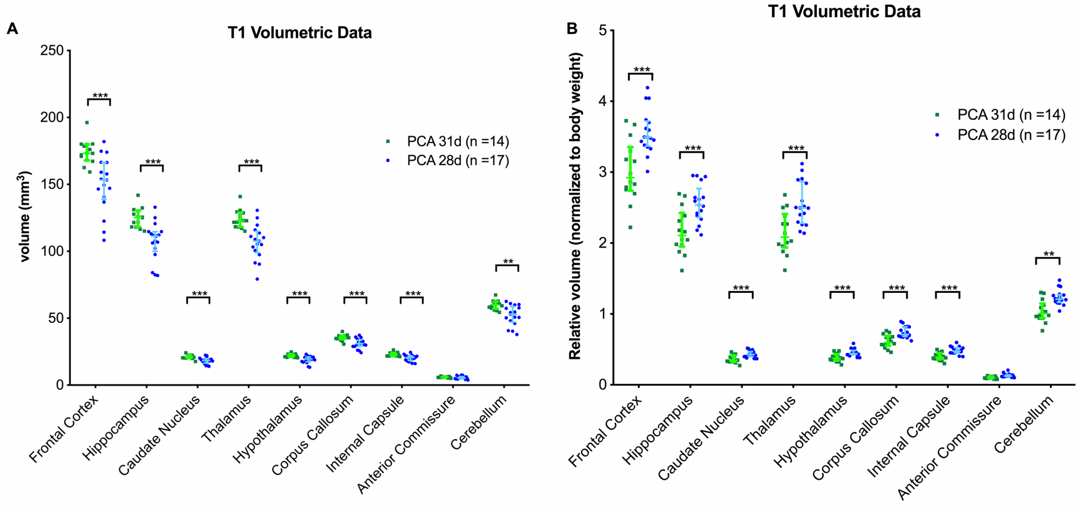

Figure 6b. T1-weighted volumetric data at postconceptional 32days. **(A)** Absolute volumes **(B)** Relative volume normalized to body weight of the animal. Data displayed as median and IQR with significance as * 0.05 ≥ p > 0.01; ** 0.01 ≥ p > 0.001; ***p < 0.001.

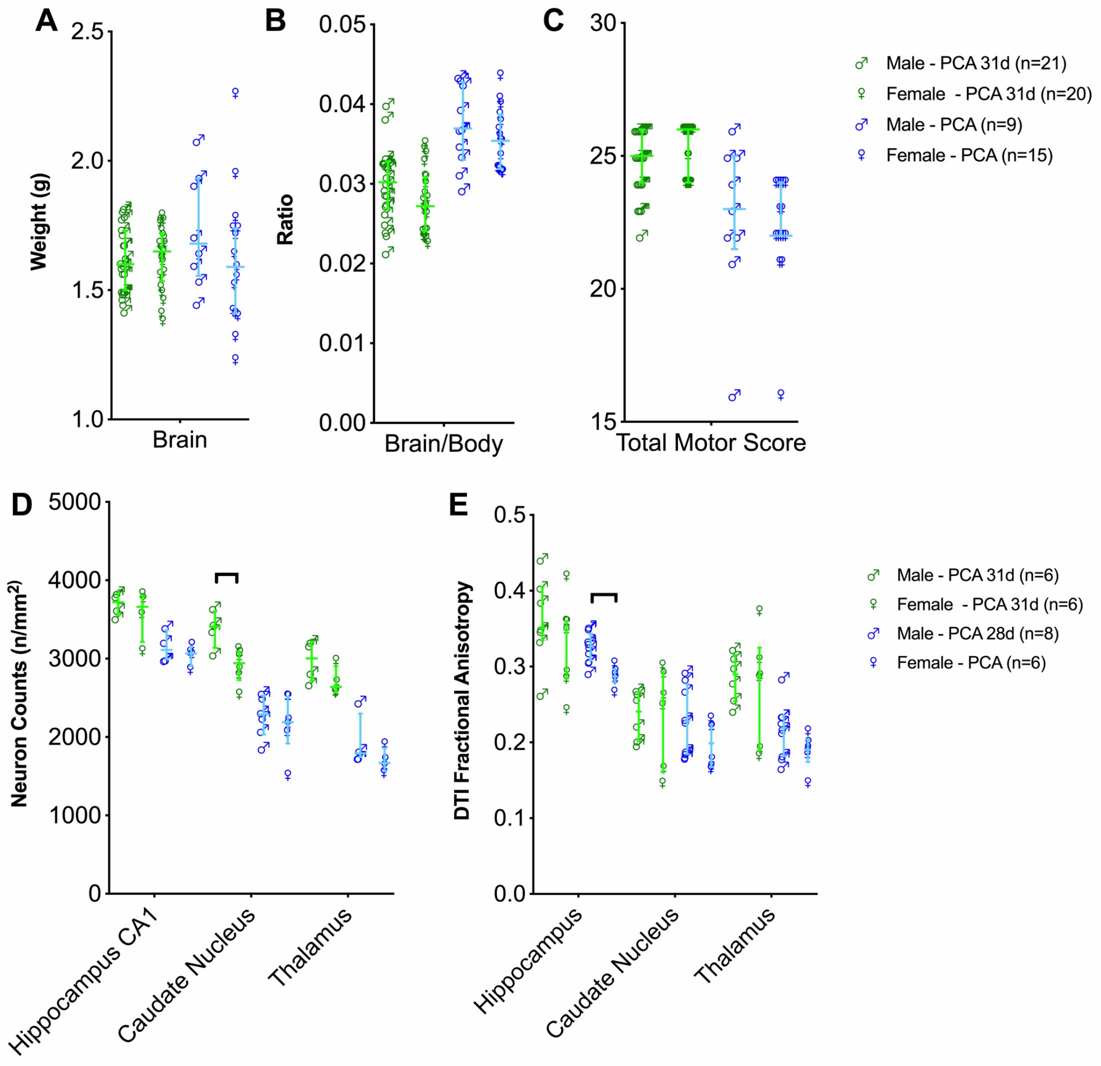

Figure 7. Sex subgroup differences in relation to the brain weight **(A)**, brain to body ratio **(B)**, motor score **(C)**, neuron densities per selected region of interest **(D)** and DTI Fractional Anisotropy per region of interest **(E)**. Data displayed as median and IQR with significance as * 0.05 ≥ p > 0.01; ** 0.01 ≥ p > 0.001; ***p < 0.001.
